# A Brief Review of Anal Cancer Screening Methods for Prevention and Earlier Diagnosis

**DOI:** 10.7759/cureus.80686

**Published:** 2025-03-16

**Authors:** Peyvand Parhizkar Roudsari, Seyedreza Mousavi, Jinous Saremian

**Affiliations:** 1 Medicine, Tehran University of Medical Sciences, Tehran, IRN; 2 Pathology, Beth Israel Deaconess Medical Center, Boston, USA; 3 Pathology, University of Florida College of Medicine-Jacksonville, Jacksonville, USA

**Keywords:** anal cytology, anoscopy, anus neoplasms, human papillomavirus, sexual behavior

## Abstract

Anal cancer has shown increasing incidence and death rates in recent years despite their lower incidence rate in the general population. Various risk factors contribute to this upward trend, with sexual risk factors playing a notable role. Additionally, there is a strong correlation between patients' survival rates and clinical outcomes with tumor stages, underscoring the importance of developing effective screening methods for anal cancer, particularly in high-risk groups. The well-established link between human papillomavirus (HPV) infection and anal tumors, combined with the success of cervical cancer screening programs, has led to some similarities in anal cancer screening strategies. However, the absence of established guidelines for anal cancer screening indicates a need for further research to assess the efficacy of these methods across different populations. Such research would enhance knowledge, awareness, and motivation for participation in screening programs. In this review, we will discuss various anal screening approaches, including their characteristics, novel biomarkers, and molecular methods, as well as prevention strategies and existing limitations in anal screening.

## Introduction and background

Anal cancer is an uncommon malignancy, accounting for 2.7% of gastrointestinal (GI) tract tumors and causing 1,670 deaths in the USA in 2022. The estimated number of new cases in females was approximately double that in males [1]. Anal cancer has shown an increasing rate of about 2.2% per year over the last ten years, which is higher than the standard historical trend observed in the 1970s and 1980s [2]. Notably, the incidence rate of squamous cell carcinoma (SCC), the major type of anal cancer, is considerably increasing, and this trend is expected to continue for the next two decades [3,4]. This makes it one of the fastest-growing carcinomas in terms of both death and incidence rates [5]. Despite the increasing incidence and mortality rates of anal cancers compared to other GI tumors [6,7], their lower incidence rate has led to less attention being devoted to screening approaches and guidelines compared to other cancers with similar characteristics, like cervical cancer [8,9]. Earlier diagnosis of anal cancers can significantly improve the quality of life for patients, as conservative approaches, including sphincter-sparing treatments, can be applied. Patients diagnosed at early stages may benefit from a five-year survival rate of more than 85% [10], whereas late diagnosis often leads to end-stage cancers with the poorest survival outcomes [11]. Indeed, survival rates are closely linked to the stage of the tumor at the time of detection [12].

The main etiologic basis of anal tumors is related to human papillomavirus (HPV) 16 and 18 infections, which have similar oncogenic roles to those in cervical cancers and have been established to cause comparable tissue changes at histologic levels [13]. These oncogenic types of HPV are involved in over 90% of anal SCCs [12]. Additionally, it has been noted that anal HPV is more commonly seen compared to cervical HPV in both HIV-infected and non-infected women in the US. The close association of HPV infection with anal tumors, along with the success of cervical cancer screening programs, has led to the development of anal cancer prevention and screening methods that are somewhat similar to those for cervical cancer [8,9,14,15]. For instance, HPV vaccination has provided adequate protection against anal cancers as well [16]. Anal cytology findings, digital anorectal examination (DARE), high-resolution anoscopy (HRA) with biopsy [17], biomarkers, and molecular tests have been found to aid in anal cancer screening, which will be discussed separately, with HPV screening playing a significant role in some of these methods [18].

Some medical conditions make individuals more vulnerable to anal cancers, including immunosuppression, HIV infection, a history of radiation to the pelvis or rectum, cervical carcinoma, and a history of HPV infection. Additionally, men who have sex with men (MSM), as well as individuals with multiple sexual partners or those who engage in receptive anal intercourse, are at a higher risk for anal cancers [19,20]. Sexual risk factors significantly increase the rate of anal cancer; for instance, HIV-infected patients are typically diagnosed about two decades earlier than non-infected individuals [21,22]. Besides the overall increasing rate of anal cancers, HIV patients are linked to a 28-fold increase in the risk of anal cancer, placing them in a higher priority group for screening purposes [23]. Anal cancer rates in HIV-infected MSM can even be compared to cervical cancer rates in women before the implementation of screening programs, underscoring the cost-effectiveness of anal screening, particularly in higher-risk groups [24,25]. In summary, given the lack of globally implemented screening programs and the necessity of early detection of anal cancers, this brief review examines various studies that have established effective screening methods.

## Review

A brief review of anal cancers

According to the World Health Organization (WHO) classification, epithelial tumors, mesenchymal tumors, and secondary tumors are the three main types of anal canal tumors. Anal intraepithelial neoplasia (AIN), Bowen disease, perianal squamous intraepithelial neoplasia, and Paget disease fall under the premalignant lesions subtype of epithelial tumors. On the other hand, SCCs, along with adenocarcinoma, verrucous carcinoma, undifferentiated carcinoma, and mucinous adenocarcinoma, form another subgroup of anal canal tumors known as carcinomas [26].

Further evaluations have led to a newer classification for AIN due to the close relationship between the underlying pathways of these cancers. In this classification, AIN I, condylomata, and atypical squamous cells of undetermined significance (ASC-US) are considered low-grade squamous intraepithelial lesions (LSILs), while high-grade squamous intraepithelial lesions (HSILs) include AIN II (moderate dysplasia) and AIN III (severe dysplasia) [27]. AIN II/III are the most advanced precancerous lesions, with a noted progression rate of 14% to cancer (malignant transformation rate) within eight years [24]. Therefore, untreated SILs can progress to malignant lesions, such as SCC [28,29], emphasizing the importance of screening programs. Early detection is crucial in preventing cancer [30].

Given the necessity of early diagnosis, common symptoms such as anal pain, bleeding, palpation of a rectal mass, and weight loss should be noted [31]. However, due to their nonspecific nature, these symptoms may lead to delayed detection and be confused with benign conditions like hemorrhoidal disorders. The primary diagnostic workup for suspected individuals includes taking a detailed patient history (including risk factors and history of anogenital lesions) and performing a proper physical and proctological examination (digital rectal exam (DRE), proctoscopy, and rectoscopy), along with histopathological and radiological assessments [32,33].

The main therapeutic options for anal cancers include surgical resection and radio-(chemo)therapy. In later stages, the focus shifts to palliative care and immuno-oncology agents. While radio-(chemo)therapy is an available approach for early stages, it may not be effective in cases of metastasis and advanced stages, underscoring the importance of early diagnosis [10,34].

Anal cancer screening approaches

There are some controversial issues regarding the recommendation of routine anal screening for AIN or SCC lesions. Many studies do not suggest routine screening, even for high-risk groups, while others have confirmed the benefits of screening [35,36]. In 2007, anal cancer screening was recommended for HIV patients with certain risk factors, including MSM, cervical or vulvar histology, and anogenital warts [30,37]. Anogenital condylomas in HIV-infected patients also place them in a higher-risk group for screening, with a higher risk for HPV-related cancers [38].

Other factors such as CD4+ counts, non-use of antiretrovirals, receptive anal intercourse, and HPV infection (especially oncogenic types) also affect the risk of AIN in HIV patients. Additionally, some studies have suggested annual anal screening for all HIV-infected patients, regardless of associated risk factors, using both anal cytology and HRA techniques [37]. Women with lower-genital tract carcinomas may also benefit from screening approaches, regardless of their HIV infection status [39,40]. Patients with autoimmune or immunosuppressive disorders, such as inflammatory bowel disease (IBD), should also be considered for screening [41].

Anal Cytology

Anal cytology has been introduced as the initial screening technique due to its low cost and wide availability, with more invasive approaches suggested for use afterward [39,42]. The histopathological similarities between the cervix and anus have raised expectations that cytological screening could reduce anal cancers, as it has in cervical carcinomas [30,35,43]. According to a study, routine anal cytology screening in HIV-positive patients may lead to a reduction in invasive anal SCC compared to those not screened [44].

In brief, anal cytology screening is a practical, simple, and painless tool that is performed blindly and has shown similar sensitivity to cervical screening, although with lower specificity. Liquid-based methods are preferred over traditional methods, mainly due to their reduced contamination, higher cellular yield, more accurate diagnosis, and higher positive predictive value [38,45]. Pap smear samples are typically collected by clinicians, but recent studies have also evaluated the efficacy of self-collected specimens, which have shown 68% sensitivity compared to 70% in clinician-collected samples. However, additional studies are needed to assess the clinical utility of this method more accurately [46].

Samples should be taken from the entire anal canal, including the transitional zone, the most common site for anal cancers. The presence of squamous metaplastic cells and rectal columnar cells indicates that the transitional zone has been sampled. Additionally, nucleated squamous cells and anucleated squamous cells in anal samples suggest sufficient sampling from both the proximal and distal portions of the anal canal [47]. Parakeratosis, perinuclear halo, and bi-/multi-nucleation are some features of reactive cells, while koilocytes and cells with nuclear atypia are indicators of AIN [43].

In detail, LSIL is indicated by degenerating koilocytes ("empty halos"), abnormal parakeratotic cells, and some irregular nuclear findings. HSIL is characterized by low-intermediate or immature metaplastic squamous cells that are smaller than the superficial and high-intermediate cells found in LSIL. Clumped chromatin, membrane abnormalities, and nuclear enlargement are typically seen in both LSIL and HSIL. High-grade cells may form clusters or appear as single cells. For detecting invasive anal SCC, clues include abnormal pleomorphic squamous cells with pyknotic nuclei; however, clinical examination should be emphasized due to the challenges in distinguishing SCC invasion from some high-grade keratinizing lesions, making the cytologic diagnosis of these lesions more uncertain. Typical invasion features may not be clearly present in invasive SCCs, which requires further sampling [48].

On the other hand, samples with atypical nuclear or chromatin characteristics that do not meet SIL criteria are categorized into two groups: atypical squamous cells of undetermined significance (ASC-US) or atypical squamous cells-cannot exclude HSIL (ASC-H). ASC-H is distinguished from ASC-US by smaller, low-intermediate, and metaplastic cells with a higher nuclear-to-cytoplasmic ratio [48].

High-Resolution Anoscopy (and Biopsy)

High-resolution anoscopy (HRA) and biopsy merit consideration after receiving abnormal cytological findings (ASC-US or higher) on anal cytology [48-50]. Since there are no established guidelines in this regard, it is suggested that all abnormal cytology results be referred to HRA [35]. Moreover, due to the reported low accuracy of anal cytology, especially in higher-grade lesions, HRA in association with anal cytology may be preferred as the first line of screening [37].

For HRA or anal colposcopy, a 3% acetic acid solution is used to saturate the epithelial layer, a disposable anoscope is required to examine the anal canal (including the keratinized area and perianal site), and a colposcope is used for microscopic evaluations [47]. HRA helps the clinician locate lesions for biopsy and further histopathologic assessments [35]. During HRA, clinicians look for vascular alterations, such as punctuation and mosaicism, which are more prevalent in HSIL. High-grade lesions commonly appear flat but can also present in warty forms. Colposcopic changes in the acetowhite layer, which are well-demarcated in both HSIL and LSIL, should also be noted during the procedure. Architectural characteristics of low-grade lesions include contour changes, such as cauliflower-like, papillary, macular, or papular features. Exophytic lesions, along with vascular alterations, may indicate malignancies, highlighting the importance of biopsy in these cases. Additionally, ulcerative or firm masses found during DRE should undergo biopsy due to the possibility of invasion [47,48].

Studies report minimal pain and 92-99% satisfaction with HRA examinations, introducing the procedure as a well-tolerated and acceptable approach in high-risk patients [51,52]. While HRA is considered the gold standard for AIN diagnosis, it is time-consuming and costly, and even some developed countries lack access to HRA-guided biopsy [39,53-55]. The quality of HRA strongly depends on the clinician's experience, requiring extensive expertise [56]. However, the higher cost-effectiveness of HRA in diagnosing AIN II/III in HIV-infected men who have sex with men (MSM) has been reported in studies. HRA may serve as an initial screening approach directly utilized in these groups, particularly when there are no geographical or resource limitations. This preference is due to the low specificity and increased false-positive results of anal cytology and HPV testing, which reduce the efficacy of these approaches [40,57].

Routine video anoscopy, along with colposcopy, offers an encouraging opportunity to provide high-quality images that can more accurately detect abnormal structures. This can increase the sensitivity of HRA and make it easier to distinguish anal lesions from related differential diagnoses [33].

Digital Anorectal Examination (DARE)

DARE is a low-cost and accessible examination that has the potential to demonstrate anal SCC at the earlier stages [38,41,58] and is usually performed during the HRA approaches, too [59]. Annual DARE has been highly recommended in high-risk groups who do not have access to HRA technologies to examine the existence of suspicious masses [60,61]. The annual DARE is a feasible, well-tolerated, and safe screening strategy for HIV-positive MSM [62,63]. DARE by patients themselves or their partners is another introduced anal cancer screening option for MSM in an article [64].

Biomarkers and Molecular Tests

HPV 16, the dominant genotype in anal precursors, has shown higher levels in AIN II/III than in AIN I, suggesting it could serve as an efficient biomarker for these lesions [18]. Additionally, mRNA detection of 14 high-risk HPV types, alongside anal cytology screening, can significantly enhance the low specificity of anal cytology alone, potentially reducing the frequency of follow-up visits. However, high-risk HPV DNA also lacks sufficient specificity since it is a common finding in HIV-infected MSM. This is similar to cervical cancer screening, where mRNA detection is often preferred over DNA-based approaches in many studies due to its higher specificity [53]. Nevertheless, according to numerous studies, DNA-based approaches may still be suitable and have shown the highest sensitivity for AIN II/III detection [65]. Consequently, the idea of co-testing high-risk HPV DNA with anal cytology has been proposed. At the next level of this algorithm, HPV 16/18 genotyping is considered for positive high-risk HPV DNA results with benign features. However, the proposed screening algorithm lacks efficient specificity despite its higher sensitivity [66,67].

Certain HPV-related biomarkers have been identified as being responsible for cellular cycle impairment. The expression of TOP2A, MKi67, and CDKN2A has been assessed, demonstrating the feasibility of exploring them in anal samples from liquid-based cytology. These biomarkers have also shown acceptable sensitivity and specificity in cervical cancer screening, indicating that they might be promising for AIN II/III screening. The molecular similarities between the cervix and anus suggest that similar cancer screening approaches and biomarkers used in cervical cancer screening may also be beneficial for anal cancer screening [18]. According to one study, p16/Ki-67 dual-stained cytology may be a useful biomarker for anal cancer screening [65]. High-risk HPV E6/E7 is another potential biomarker for anal cancer screening that requires further attention due to its proposed benefits for cervical cancer [68,69]. So far, these biomarkers (HPV E6/E7 mRNA and p16/Ki-67 dual stain) have been reported to have more sensitivity but lower specificity compared to anal cytology and HPV testing [70].

Recently, Raman spectroscopy (RESpect) has provided a new opportunity for the diagnosis and screening of AIN in the general population, and more specifically in HIV patients [71]. This non-invasive, laser-based technique works by detecting molecular changes. HRA biopsy samples can be used for RESpect investigations, which have been shown to reveal underlying SIL pathways in HIV-serodiscordant couples [44]. Furthermore, molecular risk stratification shows promise for anal cancer detection, particularly in identifying patients with a higher risk of progression. In this regard, a marker panel of ZNF582, ASCL1, and SST has been introduced, with methylation levels strongly correlating with disease severity [72].

Primary prevention strategies

The increasing incidence rate of anal cancers, along with its significant burden in high-risk groups, has drawn attention to preventive methods due to its preventable nature [73]. Given the strong correlation between HPV infection and anal cancer incidence, HPV vaccination programs offer a promising opportunity to prevent advanced lesions [74]. Additionally, addressing sexual behaviors as notable risk factors should be incorporated into more organized preventive strategies [75].

Vaccination

Vaccination has emerged in some studies as the most realistic strategy for preventing anal dysplasia [74]. The effectiveness of HPV vaccines in reducing and preventing anal cancer risk has been confirmed. In this context, the routine use of HPV4 and HPV9 vaccines has been recommended by the Advisory Committee on Immunization Practices (ACIP) [35,76,77]. The adjuvant HPV4 vaccination after HSIL treatment can reduce the anal cancer burden in some high-risk groups, according to a study by Deshmukh et al. [78]. Specifically, patients with inflammatory bowel disease (IBD) are at an increased risk for HPV-related dysplasia, with a 28-fold higher incidence risk for AIN. A related case report has confirmed the safe use of the inactivated HPV4 vaccine regarding immunologic reactions. Consequently, women with IBD appear to benefit particularly from HPV vaccination [79].

Changing High-Risk Behaviors

Many anal cancer-associated risk factors are related to lifestyle and behavioral parameters, particularly sexual habits. In this context, individuals who engage in receptive anal intercourse, have condomless contact, have multiple partners, or have a history of cigarette smoking are at a higher risk for anal cancers, highlighting the need for awareness among MSM [80-82]. The transitional zone, which is the primary site for metaplasia, involves the metaplastic transformation of columnar epithelium into squamous cells, a process influenced by anal intercourse. Indeed, impairment of the transitional zone's normal tissue can facilitate HPV infection, illustrating the impact of anal penetration on HPV infection and, consequently, on anal cancers [48]. Convincing data support the association between anal intercourse and oral-anal contact with HPV infection persistence, which can lead to anal cancers. This underscores the importance of consultations about primary prevention measures, such as condom use and vaccination, for high-risk groups [75].

**Figure 1:**
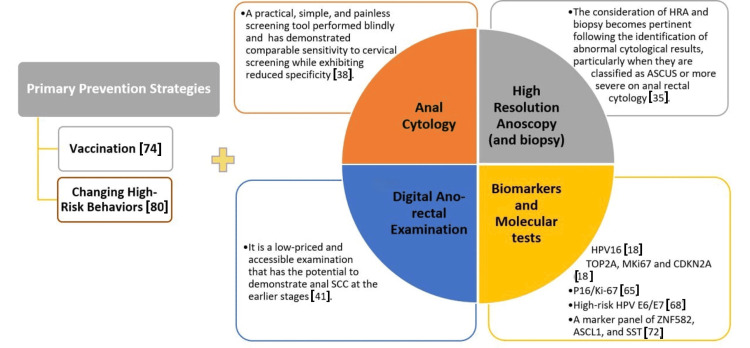
Primary prevention strategies and approaches to anal cancer screening [18,35,38,41,65,68,72,74,80,83] Primary prevention strategies and anal cancer screening approaches are closely interconnected with the need to disseminate more extensive information to high-risk populations. It is essential to provide these populations with comprehensive information about HPV/HIV infection and other risk factors. This increased awareness can enhance their understanding of the importance of anal cancer screening and preventive measures, especially in the absence of established guidelines. ASC-US: typical squamous cells of undetermined significance; HIV: human immunodeficiency virus; HPV: human papillomavirus; HRA: high-resolution anoscopy; SCC: squamous cell carcinoma The image is created by Peyvand Parhizkar Roudsari

These two approaches are summarized in Figure 1. 

Limitations and future directions

There are still several challenges in anal cancer screening, particularly within the MSM group. These challenges include stigma and embarrassment related to screening, fear, physical and psychological distress, and unawareness, which can lead to depression and isolation and deter individuals from participating in screening programs. Therefore, it is crucial to provide high-risk populations with more information about HPV/HIV infection and other risk factors to increase their understanding of anal cancer screening and preventive measures, especially given the lack of established guidelines [83,84].

JM Palefsky has noted that addressing certain challenges is necessary for the acceptance of routine anal screening among high-risk individuals. In this regard, the effects of HSIL treatment on reducing anal cancer incidence should be further investigated, and current screening methods and treatments for HSIL should be optimized. Preventive methods are highly recommended in areas with significant numbers of high-risk populations [60]. Global protocols could help define the best timing and methods for screening and identify the target population [85]. However, due to the absence of global protocols and limited data, systematic reporting may face challenges, making it harder to support follow-up programs [86]. The reported low level of concern about anal cancer highlights the need to increase public awareness and motivation to adhere to screening recommendations by emphasizing the importance of routine screening [87].

The benefits of anal cancer screening should be further explored in future research, similar to previous studies in cervical cancer screening that demonstrated associated benefits. Additionally, existing approaches should be compared in further studies to determine the optimal strategy for anal cancer screening in vulnerable groups. High-risk groups, such as HIV-infected patients and immunosuppressed individuals, are expected to experience higher survival rates in the future, indicating a greater need for screening for HPV-related complications like anal cancers due to increased exposure. Beyond the HIV-infected MSM population, other high-risk groups should also be considered for anal cancer screening, as most literature has focused on HIV-infected MSM [40,88-98].

Recent advancements in anal cancer screening include systematic analyses of genome-based studies to identify key driver gene expressions involved in anal squamous cell carcinoma and their associated signaling pathways [99]. Additionally, research has highlighted the role of the microbiome, revealing that elevated levels of microbiome-derived proteins, such as those involved in succinyl coenzyme A and cobalamin production, are significantly linked to HSILs. These findings offer new insights into the molecular mechanisms underlying anal cancer progression and may contribute to improved screening strategies [100].

## Conclusions

Although anal cancer is a rare condition, its incidence and mortality rates have been gradually increasing in recent years. Therefore, it is becoming more important to consider screening and prevention strategies in the diagnostic field. Certain conditions that elevate the risk of anal cancers require increased attention for screening purposes. HPV vaccination as a primary prevention approach appears to be effective in reducing the rising incidence of anal cancer. Given the significant role of sexual behaviors in anal cancer development, protective measures, particularly in high-risk groups, should be emphasized. Various approaches have been introduced to diagnose cancerous lesions at earlier stages, especially for high-risk individuals, such as anal cytology, HRA and biopsy, DARE, biomarkers, and molecular tests, all of which have shown promising results. However, there remains a lack of knowledge and awareness about the targeted populations and the effectiveness of these screening tools, highlighting the need for well-defined guidelines to underscore the importance of routine screening.
